# 使用气道内杂交支架治疗复杂气道狭窄和气道瘘疗效与安全性分析

**DOI:** 10.3779/j.issn.1009-3419.2020.104.05

**Published:** 2020-06-20

**Authors:** 愉 陈, 子青 周, 家欣 冯, 长镐 钟, 丽雅 卢, 小波 陈, 纯丽 唐, 时悦 李

**Affiliations:** 510120 广州，呼吸疾病国家重点实验室，广州医科大学附属第一医院，广州呼吸健康研究院 State Key Laboratory of Respiratory Disease, National Clinical Research Center for Respiratory Disease, Guangzhou Institute of Respiratory Disease, First Affiliated Hospital of Guangzhou Medical University, Guangzhou 510120, China

**Keywords:** 杂交支架, 恶性狭窄, 气管食管瘘, 复杂气道, Hybrid stents, Malignant stenosis, Tracheoesophageal fistula, Complex airway

## Abstract

**背景与目的:**

气道内支架广泛应用于气管狭窄和气管瘘的治疗，但使用气道内支架重建复杂气道的临床数据仍不充足。硅酮支架杂交金属支架重建复杂气道的有效性和安全性。

**方法:**

纳入无法手术的复杂恶性气道狭窄和气道瘘患者。使用Y型硅酮支架联合金属覆膜支架（杂交支架）重建气道。评价置入支架后6个月的疗效和并发症。

**结果:**

共纳入23例患者，置入23枚Y型硅酮支架和25枚金属覆膜支架。19例患者（19/23, 82.6%）置入支架后症状迅速缓解。支架平均置入（153.43±9.14）d。置入支架后改良呼吸困难指数（modified British Medical Research Council, mMRC）、卡氏功能状态（Karnofsky performance status, KPS）评分和功能状态（performance status, PS）评分显著改善。12例患者带支架生存超过6个月。其余患者肿瘤进展导致6个月内死亡。无支架置入相关死亡及严重并发症。

**结论:**

杂交支架重建恶性复杂气道疗效确切，耐受良好。

## Abstract (en)

**Background and objective:**

Airway stents has been widely used in airway stenosis and fistula, yet clinical date of airway stents in reestablishment a complex airway is insufficient. The aim of this study is to evaluate the efficacy and safety to combine the silicon stent and the metal stent in reestablishment a complex malignant airway.

**Methods:**

Patients with non-operable complex malignant airway stenosis and fistula were recruited in this study. Silicon Y stent combined with covered metal stent (Hybrid stent) were inserted to reestablishment the airway. Clinical outcomes and complications were observed over six months.

**Results:**

A total of 23 silicon Y stents and 25 covered metal stents were inserted in 23 patients. Nineteen of 23 (19/23, 82.6%) patients felt a immediately relieving of current symptoms. The mean duration of stents placement in patients was (153.43±9.14) days. The modified British Medical Research Council, Karnofsky Performance Status and Performance Status scale were improved significantly after stenting. A total of 12 patients living with stent at 6 months. Others dead of tumor progression. There were no death or immediate complications related to hybrid stenting implication.

**Conclusion:**

Hybrid stenting proved to be useful and was well-tolerated in the management of complex malignant airway stenosis and fistula.

气管狭窄和气管食管瘘是恶性进展期肿瘤的严重并发症，大多数由肺癌导致。食管癌、甲状腺癌、纵隔肿瘤和肿瘤特异性治疗（如：放疗）同样可以引起气管、支气管食管瘘、气管狭窄。气管狭窄主要引起气流受限，狭窄直径小于5 mm可导致呼吸窘迫^[1]^。气管食管瘘会导致反复误吸、咳嗽和感染。这些并发症显著降低患者生活质量，影响预后并增加死亡率。而且进展期恶性肿瘤患者往往缺少肿瘤治疗机会和手术机会。因此，姑息治疗成为这部分患者重要的缓解症状的治疗手段^[2]^。

介入支气管镜技术已经广泛的应用于不能手术的进展期肿瘤患者的减症治疗或姑息治疗中^[2-4]^。气道支架是气道狭窄和气道瘘最常用的治疗手段^[2]^，它能保持气道通畅，缓解气道狭窄、气道瘘导致的一系列症状^[2-6]^。是终末期肿瘤患者的有效姑息治疗手段。此外，气道支架还可以为患者争取时间接受进一步肿瘤针对性治疗^[7]^。Chris^[4]^报道了硅酮支架缓解恶性狭窄呼吸困难有效率达90%。Miyazawa等^[3]^的研究发现82%的患者放置金属支架后症状迅速缓解。Dutau等^[2]^报道了Y型硅酮支架对于侵犯隆突的恶性狭窄和气管食管瘘有较好的疗效。但这些研究纳入的患者均为简单狭窄，病变范围较小，单一支架可覆盖病变部位。较少有研究报道气道支架治疗复杂狭窄（狭窄长度较长或狭窄合并气道瘘）。本研究将探索硅酮支架联合金属支架重建复杂气道的安全性和有效性。我们定义硅酮支架联合金属支架为“杂交支架”。

## 1 对象和方法

### 1.1 对象

纳入2016年8月-2019年8月在广州医科大学附属第一医院诊断为恶性气道狭窄或气道瘘的患者。杂交支架置入的适应证：①无法手术治疗的恶性气道狭窄或气道瘘；②气道狭窄程度大于50%，患者有显著呼吸困难，肺不张或狭窄导致的肺炎；③复杂狭窄，狭窄累积隆突，同时累及气管或主支气管，或合并气道瘘；④巨大瘘口，瘘口部位在隆突附近。排除标准：①简单狭窄/气道瘘，预计可以通过单个支架治疗；②早期气道肿瘤预计可以通过手术治疗。研究方案通过广州医科大学第一附属医院伦理委员会批准（伦理号：2018-16）。

**1:** 患者基本特征 Patients' characteristics

Patient characteristics	Data
Gender	
Male	15
Female	8
Age (yr)	53.78 (47-59)
Minimum	30
Maximum	78
Diagnosis	
Lung primary tumors	10
Esophageal tumors	10
Metastatic tumors	3
Stents implement	48
Silicon Y stents	23
Covered metal stents	25

### 1.2 操作过程

治疗策略由3名有经验的介入肺脏病医生制定。患者术前进行三维重建CT和常规支气管镜检查评估气道情况，包括狭窄长度、瘘口面积、与正常气道的比邻关系。使用高压扩张球囊加压至特定球囊外径于狭窄处上下移动确定狭窄处直径。本研究纳入病例病变累及气管、隆突和主支气管，使用Dumon Y型硅酮支架联合直筒金属支架治疗。

根据气道的直径和病变长度定制支架。对于Y型硅酮支架，气管支和主支气管支的直径设定根据三维CT重建测量的正常气道内径。支架长度超过病变长度，以达到覆盖病变两端超过10 mm。如果主支气管病变长度距离隆突超过20 mm，将Y型支架支气管支的长度设置为15 mm，并叠加另一个Ultraflex带膜金属支架覆盖病灶。金属支架的内径设置同Y型支架，金属支架长度覆盖左右主支气管。

患者在全麻下插入硬质支气管镜，在高频通气和常频通气叠加下完成支架置入操作。用合适的方法如激光、电刀和球囊扩张等清除气道内肿物或病变，使气道复通。使用推送法或回退法置入Y型硅酮支架。对于合并瘘的患者，将硬镜远端送至完全覆盖瘘口，并使用回退法置入支架。当Y型硅酮支架的支气管支送入一侧主支气管后，使用鳄鱼钳钳夹硅酮支架缓慢拉出支架直到另一支滑入对侧主支气管（通常是较短支），再向前推送并使用球囊扩张使支架贴合隆突并贴合病变^[2]^。随后，使用导丝引导置入另一个金属支架，并用钳子和球囊调整金属支架使杂交支架完全覆盖病变。

### 1.3 杂交支架的有效性和安全性评价

置入支架后24 h复查胸片排除手术导致的纵隔气肿和气胸。患者置入杂交支架后的第1天、7天、30天和180天返院，支气管镜复查支架位置和通畅度。当出现咳嗽、气促和气短等不适时提前返院复查。记录支架相关症状和不良反应。

使用mMRC评分评价支架置入后肺功能改善情况。mMRC评分分级：0级：上楼梯无临床症状；Ⅰ级：上楼梯可出现呼吸困难；Ⅱ级：步行100米出现呼吸困难；Ⅲ级：轻微气力活动即出现呼吸困难症状（说话，穿衣等）；Ⅳ级：平卧或休息时即有呼吸困难症状^[3]^。使用卡氏体力状态评分（Karnofsky performance status, KPS）评分置入支架前后生活质量改善情况。

### 1.4 统计分析

使用SPSS 16.0版本进行统计学分析（SPSS Inc., Chicago, IL, USA），所有数据使用*Kolmogorov-Smirnov*检验。正态分布变量使用均值±标准差表示，非正太分布变量使用中位数（四分位间距）表示。配对资料使用配对*t*检验比较大小。组间差异使用独立样本*t*检验比较大小。*P* < 0.05认为有统计学差异。

## 2 结果

### 2.1 人口学资料

研究共纳入23例患者（12例男性，11例女性），平均年龄53.8岁。11例患者气道病变为狭窄，无合并瘘，其中10例诊断为肺癌，1例诊断为食管癌，另外3例诊断为转移性癌。9例患者存在气道瘘，诊断为食管鳞癌，其中5例存在瘘口合并狭窄。病变分布如下：20例（86.9%）存在气管受累，12例（52.1%）存在隆突受累，16例（69.6%）存在左主支气管受累，11例（47.8%）存在主支气管受累（图 1）。总共置入23枚Y型硅酮支架和25枚覆膜金属支架。

**1:**
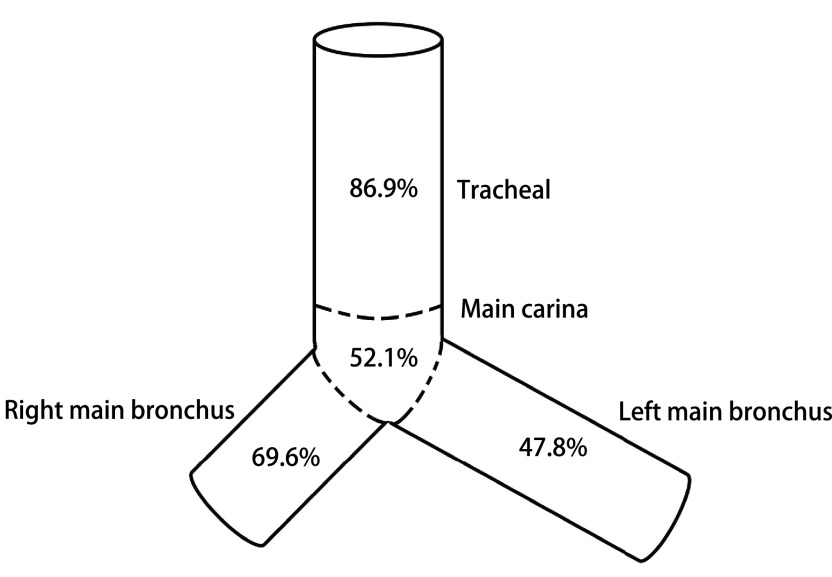
23例患者气道病变位置分布 Lesion distribution of the airway in 23 patients

### 2.2 临床疗效

所有支架均可使阻塞的管腔复通，并完全覆盖气道瘘口。置入支架后，15例（15/19, 78.9%）存在狭窄的患者呼吸困难症状迅速缓解。9例合并气道瘘的患者中，9例呛咳症状缓解。所有合并瘘的患者7 d-9 d后肺部感染得到控制，其中1例可正常进食。2例患者第7天和第62天失访，支架平均置入时间为（153.43±9.14）d。其中1枚金属支架置入后151 d因为瘘口再通移除支架，并更换心脏封堵器封堵瘘口。1枚Y型支架因为管腔再狭窄移除，重新放置一枚更小的Y型支架后管腔再通。共有12例患者带支架存活超过6个月。其余患者死于肿瘤基础疾病。支架放置后疗效情况见表 2和表 3。

**2:** 9例气道瘘患者放置支架情况和临床结局 Stents and clinical outcomes of patients with fistula

No./ Age, yr/Gender	Primary tumor	Location (area, cm^2^)	Combined with stenosis	Type of Y stent^*^	Type of metal stent^**^	Duration (d)	Outcome (d)
1/67/male	Esophageal squamous cancer	Distal left main bronchus (1*1)	No	50-10-30/18-14-14	14-40	> 180	Removed in 151 day and inserted a occluder
2/56/male	Esophageal squamous cancer	Lower tracheal (0.5*0.5)	Left main bronchus	55-15-10/18-14-14	12-30	> 180	Living with stents (> 180)
3/54/male	Esophageal squamous cancer	Lower tracheal (1*0.5)	Right main bronchus	50-15-30/16-13-13	14-40	> 180	Living with stents (> 180)
4/47/male	Esophageal squamous cancer	Lower tracheal and left main bronchus (3*1.5)	No	45-10-15/16-13-13	10-40	> 180	Living with stents (> 180)
5/46/male	Esophageal squamous cancer	Lower tracheal and left main bronchus (1*1)	No	45-10-30/16-13-13	14-40	7	Lost
6/68/male	Esophageal squamous cancer	Lower tracheal and right main bronchus (2.5*1)	No	50-25-30/18-14-14	14-40	92	Dead
7/49/male	Esophageal squamous cancer	Lower tracheal (2*2.5)	Right main bronchus	50-15-35/18-14-14	14-40	91	Living with stents (> 90)
8/66/male	Esophageal squamous cancer	Right main bronchus (1*1)	Right mainstem	55-15-10/18-14-14	10-30	62	Lost
9/45/male	Esophageal squamous carcinoma	Left main bronchus (1*1)	Left main bronchus	45-15-10/16-13-13	12-40	151	Dead
^*^Type of Y stents are presented as length of tracheal body-left limb-right limb/diameter of tracheal body-left limb-right limb. ^**^Type of metal stents are presented as diameter-length.							

**3:** 11例狭窄患者放置支架类型和临床结局 Stents and clinical outcomes of patients with stenosis

No./ Age, yr/Gender	Primary tumor	Location	Type of Y stent^*^	Type of metal stent^**^	Duration (d)	Outcome (d)
10/50/male	Large cell lung cancer	Main carina and both mainstem bronchi	40-25-15/ 18-14-14	12-30	151	Dead
11/55/male	Squamous lung cancer	Main carina and left main bronchi	40-15-15/ 18-14-14	14-40	65	Dead
12/54/male	Tracheal adenoid cystic carcinoma	Main carina and both mainstem bronchi	45-25-20/ 16-13-13	12-40	> 180	Living with stents (> 180)
13/49/female	Small cell lung cancer	Main carina and both mainstem bronchi	40-25-15/ 15-12-12	12-30	152	Dead
14/55/female	Lung adenocarcinoma	Tracheal, main carina and left main bronchus	35-20-10/ 15-12-12	12-40	> 180	Living with stents (> 180)
15/78/female	Squamous lung cancer	Tracheal, main carina and right main bronchus	110-15-30/ 18-14-14	10-20	> 180	Living with stents (> 180)
16/50/male	Squamous lung cancer	Tracheal, main carina and left main bronchus	60-20-10/ 16-13-13	12-40	> 180	Living with stents (> 180)
17/30/female	Squamous lung cancer	Tracheal, main carina and left main bronchus	30-15-15/ 15-12-12	12-40	62	Dead
18/59/male	Esophageal squamous cancer	Tracheal, main carina and right main bronchus	50-25-15/ 16-13-13	10-30	> 180	Living with stents (> 180)
19/63/female	Neuroblastoma	Tracheal and both mainstem bronchi	75-15-15/ 15-12-12	12-40	93	Dead
20/45/male	Liver cancer	Main carina and both mainstem bronchi	30-15-15/ 18-14-14	12-40	> 180	Living with stents (> 180)
21/50/female	Breast cancer	Tracheal, main carina and both mainstem bronchi	85-15-15/ 15-13-13	12-30	> 180	Living with stents (> 180)
22/45/female	Squamous lung cancer	Tracheal and left main bronchus	55-15-30/ 15-12-12	12-40	95	Dead
23/56/female	Lung lymphoepithelioma-like carcinoma	Tracheal, main carina and left main bronchus	60-10-15/ 16-13-13	12-30	> 180	Living with stents (> 180)
^*^Type of Y stents are presented as length of tracheal body-left limb-right limb/diameter of tracheal body-left limb-right limb. ^**^Type of metal stents are presented as diameter-length.						

合并狭窄的19例患者放置支架前平均mMRC评分为（3.43±0.51）分，放置支架后显著降低到（2.91±0.51）分（*t*=3.46, *P*=0.01）。22例患者（22/23, 95.6%）KPS评分显著改善，从术前（46.95±15.79）分增加至（62.17±10.42）分（*t*=3.86, *P*=0.01）。9例患者放置支架前PS评分大于3分，其中7例（1例合并气道瘘，6例单纯狭窄，7/9，77.8%）放置支架后PS评分显著改善，从（3.3±0.73）分降低到（2.21±0.51）分（*Z*=-2.30, *P*=0.02）。对于放置支架前PS评分小于3分的病例，放置支架后PS评分无显著改善。

### 2.3 并发症

无手术操作相关短期并发症（如：出血、气胸和纵隔气肿等）。4例患者60 d内发生支架内肉芽增生，但不影响支架通畅度，予钳除。9例病例出现分泌物潴留，予吸除后嘱加强雾化后分泌物潴留改善。随访期内无支架移位发生。无支架相关严重并发症及死亡发生。

**2:**
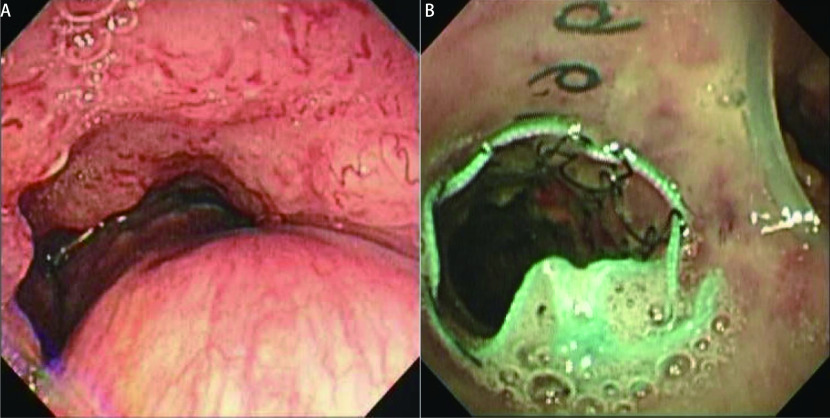
病变累及气管下段、隆突和左主支气管，置入杂交支架完成气道重建。A：置入支架前；B：置入杂交支架后。 Distal tracheal, carinal, and proximal left main bronchus involvement before and after the hybrid stent placement. A: Before stenting; B: After hybrid stent placement.

## 3 讨论

Ultraflex覆膜金属支架和Dumon硅酮支架已经广泛的在临床中用于治疗恶性狭窄和恶性气道瘘。但较少有研究报道联合两种支架（杂交支架）处理复杂的恶性狭窄/气道瘘。本研究使用杂交支架对复杂恶性狭窄、气道瘘进行气道重建。结果表明，杂交支架重建恶性复杂气道可维持阻塞气道的通畅，封堵瘘口，患者耐受好，并发症少。对于恶性复杂狭窄、气道瘘的气道重建展现出较好的疗效和安全性。

杂交支架置入的适应症是需要气道重建但无法耐受手术治疗的复杂气道病变，特别是恶性气道病变。本研究中，杂交支架治疗了14例侵犯隆突的复杂狭窄，4例巨大瘘口，5例狭窄合并瘘患者。这些患者的特点是气道病变复杂，狭窄长度长，瘘口较大或位置特殊，难以用单一支架解除狭窄梗阻或封堵瘘口。因此，使用Y型硅酮支架，联合覆膜金属支架对这些患者的气道进行重建。78.6%合并狭窄的患者呼吸困难症状迅速缓解。同时，与气道瘘相关的感染在放置支架后均得到控制。杂交支架置入对症状的缓解与以往Y型硅酮支架和覆膜金属支架的研究相似^[2, 3]^。对于此类复杂气道病变且一般情况较差的患者，考虑到操作安全性，对是否使用支架治疗存在争议^[2, 3, 8, 9]^，尽管如此，我们认为，如果有熟练的内镜操作经验，使用杂交支架治疗是有效的方法。支架中位放置时间为103 d，与既往研究中此类患者的平均生存时间相似^[2, 10]^，与治疗方法无关，包括外科和内镜治疗。但是，保守治疗效果并不好，因为这类患者容易发生致命的气道阻塞和感染^[5]^。既往研究对于支架治疗恶性气道狭窄或气道瘘PS评分改善的报道结果不一^[7, 11]^。本研究中78%的患者（7/9）PS评分显著改善，因为这些患者一般情况的恶化是由气管狭窄或气道瘘导致。对于这类放置支架改善PS评分的患者，可进一步尝试肿瘤针对性治疗。即使患者对肿瘤治疗反应不好，杂交支架仍然可以达到缓解这部分患者临床症状的效果。本研究病例中最常见的并发症是轻度肉芽增生和分泌物潴留，6个月内无严重并发症发生。提示杂交支架技术对于恶性气道狭窄短期疗效较好，患者耐受良好，并发症少。

因为隆突的解剖结构呈Y型，直筒支架在隆突病变的患者中使用受到限制^[1]^。Y型支架包含了气管支和两个支气管支，更贴合隆突解剖结构，所以对于Y型硅酮支架发生支架移位的概率很低。相比金属支架或其他直筒型支架，Y型支架可在带来更大径向支撑力的同时减少对管壁的摩擦和刺激^[12, 13]^。因其组织相容性好，不会卡顿在气道内无法取出。当患者对肿瘤治疗反映较好，气道内肿瘤消褪后，可考虑取出支架^[2, 13]^。因此，Y型硅酮支架特别适合于累及气管下段、隆突和左右主支气管的恶性气道狭窄、气道瘘^[1, 2]^。但Y型硅酮支架的放置也有缺点，首先，需要全麻下经硬镜置入，而且对操作者操作经验要求较高^[2, 4, 7]^。因为硅酮支架顺应性较差，对于特殊病变硅酮支架容易在气道内成角，导致再狭窄发生。且由于主支气管内径近段较大，外周端较小，硅酮支架并不适合放置于靠外周的气道。相比之下，金属支架延展性更好，对于复杂形状的气道或远端中央气道病变，金属支架能更好地贴合气道达到气道重建^[3]^，且可以很容易通过硬镜或可弯曲支气管镜放置。对于合并隆突和远端主支气管病变的患者，我们推荐使用叠加一枚覆膜金属支架使之更贴合远端气道而达到气道重建^[2]^。金属支架中间覆膜可减少肉芽增生，两端7.5 mm部分无覆膜，可促进支架上皮化，从而改善纤毛清除能力，减少支架移位。杂交支架覆盖范围较广，从气管到主支气管远端，由于支气管部分叠加金属支架，支架与气道贴合度较好，可减少支架成角并进一步减少支架移位。对于各种复杂情况，如狭窄长度较长、不同部位狭窄直径不同，均可尝试使用杂交支架重建气道。但应该注意的是杂交支架由于覆盖气道面积较大，痰液潴留风险增大。所以我们建议患者每天3次-5次生理盐水雾化稀释痰液。对于存在隆突狭窄合并特定部位的气道瘘（如上叶支气管胸膜瘘），也可以尝试Y型支架联合心脏封堵器重建气道^[14, 15]^。

本研究中病例术中使用高频通气和常频通气两种通气方式，可达到减少漏气，降低二氧化碳潴留，减少手术时间的效果。置入支架前，硬质支气管镜远端应伸入到病变的远端，放置前应清楚辨认左右主支气管位置，确保Y型硅酮支架左右主支正确放入左右主支气管。对于合并瘘的病例，应使用回退法放置支架，避免推送支架时对瘘口造成二次损伤。金属支架的近端连接Y型硅酮支架支气管支的远端，可增加杂交支架的稳定性。我们建议Y型支架的内径应该与气管和左右主支气管内径匹配，不建议选择过大内径的支架，内径过大会压迫气道，导致气道损伤，延迟修复。内径过小也是不适合的，会带来支架松动和成角。Y型支架的支气管支设置为超过主支气管隆突开口平面15 mm，作用是维持隆突结构减少位移，但不应超过30 mm，因为过长的支气管支容易导致支架成角。对于主支气管病变较长的，Y型硅酮支架支气管支叠加金属支架可以很好的避免支架成角发生。

本研究纳入的患者均为终末期累及气道的恶性肿瘤患者，放置杂交支架的目的是缓解症状而不是彻底治愈。研究缺少长期随访数据。在6个月的观察期内并无研究并发症发生。但杂交支架长期的有效性和安全性仍需进一步研究探索。尤其是对于良性病变，包括复发性多软骨炎、气管支气管结核和创伤性气道狭窄、气道瘘。

综上所述，杂交支架技术对终末期恶性气道病变的姑息治疗和气道重建是值得推荐的，患者狭窄、气道瘘相关症状缓解明显，生活质量改善，并发症较少。
